# The Economy of the Animal Kingdom Considered Anatomically, Physically, and Philosophically

**Published:** 1846-10-01

**Authors:** 


THE ECONOMY OF THE ANIMAL KINGDOM CONSIDERED ANATOMICALLY, PHYSICALLY, AND PHILOSOPHICALLY. By *Emanuel Swedenborg*, late Member of the House of Nobles in the Royal Diet of Sweden, &c. Translated from the Latin by the Rev. *Augustus Clissold*, M.A. Two Volumes 8vo. London: W. Newberry, 1846.

SWEDENBORG, or Swedberg, as he was named before he was ennobled in 1719, is known, for the most part, as a well-meaning enthusiast, and as the founder of the religious sect named after him, "*Swedenborgians.*" He was, however, one of the most distinguished philosophers of the age in which he lived; well versed in almost the entire circle of science; and, moreover, a practical anatomist. It has been affirmed that he had even anticipated Dalton, not only as to the atomic theory itself, but likewise as to some of its details. His scientific works are very numerous, and treat of some of the deepest and most fundamental truths; it was, indeed, the object of Swedenborg to systematize the vast accumulation of former ages, to select the leading truths from the various schools of philosophy, and to establish the principles which flow from them. To undertake at all a task thus vast and comprehensive, indicates the possession of the most varied information; that the effort was successful would not be expected by those who recal the state of knowledge at the period, the first half of the last century, when this remarkable man composed his scientific works. It is, however, interesting to observe, as an indication of the complexion of Swedenborg's mind, that he himself was convinced the time was ripe for the bold enunciation of the broadest principles in every department of science, including the most abstruse questions in physics, chemistry, and physiology. "In approaching the human body," we here borrow a passage from the interesting biographical sketch contained in that valuable work, the Penny Cyclopædia, "he again insisted on the necessity for principles and generalizations, without which, he said, 'facts themselves would grow obsolete and perish:' adding that 'unless he were much mistaken, the destinies of the world were leading to this issue.'" Even in these physiological inquiries he entered into metaphysics, being resolved thoroughly to examine "the world or microcosm which the soul inhabits, in the assurance that she should be sought for no where but in her own kingdom." It was about the time when the last of these scientific works were published, namely in 1745, that their author conceived he had received a divine revelation; and those who carefully compare his theological with his previous philosophic speculations, will find in more than one respect the same train of thought running through them ; as if his conviction that the time was come for proclaiming the ultimate principles of all science, had led to his notion that the second advent of the Lord and the Apocalyptic New Jerusalem were truly realised and witnessed by him.

A philosophic work like that before us, written by a man of such marked psychical peculiarities as Swedenborg, cannot fail to interest all who have leisure to peruse a production which, having been long since superseded by later and more sound writers, has lost all practical utility. It has indeed puzzled us to comprehend the motives of the learned and Reverend translator in bringing again into light a treatise, which has become well nigh obsolete. That no scientific views have had influence, we must infer from the omission of those copious notes, by which alone the multiplicity of errors long since exploded, but which pervade the whole work, could have been corrected and explained. As it is we have, mixed doubtless with much sterling matter, a multitude of fanciful hypotheses and details concerning the "animal spirits," "spirituous fluid," and the "corcula," or little hearts destined for their circulation, consisting of "the spherules of the cineritious substance;" the whole clothed in a language, which, owing to the advance of physical, chemical and physiological science, has to a great extent lost its significance. The following extract relating to the composition of the blood and to the "spirituous fluid," will enable our readers to judge of the general style of the work.

"Blood comprehends in every one of its spherules, mere first principles, elements, and simples. Consequently it possesses potentially and virtually every single thing in the mundane system which is producible from first principles, elements or simples; that is, everything which is possible. Those volatile ethereal substances which temper the spirituous fluid, are the first and only entities of their own and the following degrees; hence also they are the elements of those degrees. The volatile aërial substances are also the simples of their own and the next following degree; while the saline cube, which is the cement of the whole part, is the simple of its own degree." Vol. 1, p. 76.

With respect to the "spirituous fluid" it is described as consisting of a subtle matter poured into the nervous fibres, from the blood-vessels, in the cineritious substance of the brain and spinal cord, and which, after circulating through the nerves, returns again into the vessels. The hypothetical notions of Swedenborg respecting the circulation generally, and the movements of this subtle fluid, are thus explained :—

"In the vessels, equally as in the blood and membranes, there are three degrees of composition to be taken into consideration, all of which should be distinctly perceived. The vessels of the first degree are those commonly called blood-vessels ; the vessels of the second degree are the exsanguious vessels ; and the vessels of the third degree are the fibres of the nerves. In conformity with these various degrees of vessels, the circulation itself is subtriplicate; namely first, a less universal circulation, which is that of the red blood ; secondly, a more universal circulation, which is that of the purer blood ; and thirdly, a most universal circulation, which is that of the spirituous fluid.

"While the red blood is passing from vessels of its own order into vessels of another order, it becomes divided into the purer blood, or into blood of the second order ; the saline, urinous, or sulphurous atoms which had entered into the composition of that degree, being deposited at the mouths of ingress or division. A

corresponding operation is carried on when the blood passes from vessels of the second order into vessels of the first, or into the fibres.

"After reaching the fibres, the blood continues its passage through them, returns into the vessels of the second and third orders, and becomes again compounded by passing through degrees similar to those by which it had become divided." Vol. 1, p. 98.

All this is mere surmise, and much of it is entirely disproved by the exact knowledge possessed in the present day with regard to the arrangement of the blood-vessels and nerve-tubes. Those who are accustomed to minute observation, are satisfied that the limits of the vascular system are definitively established ; they are perfectly recognisable, and demonstrate that there is no such relation as that here surmised with the nerve-tubes. If it be argued that although Swedenborg was in error as to the path by which the "spirituous fluid" flowed from the blood, and that the transit may be effected as well by endosmose as by direct channels, it may be replied that suppositions of this kind, resting upon no ascertained facts, have abounded in every department of science, but that they are not the means by which truth has ever been elicited.

The volumes before us contain, as we have said, much valuable matter, consisting however, for the most part, of extended extracts from the works of Malpighi, Leeuwenhock, Morgagni, and indeed from most of the distinguished authorities of the 17th and 18th centuries ; many of Swedenborg's own observations also afford interesting subjects for reflexion.

His account of the ganglionic or nervous corpuscles, as they are termed, will be read with interest by those who are acquainted with the latest descriptions which have appeared of these important elements of the gray matter.

"The cortical substance, either when lying proximately beneath the pia mater, and watered, nourished and cherished by the purer blood, or when, under the name of the cineritious substance, it occupies various tracts more remote from the surface, may, by the naked eye, and more plainly still by the help of glasses, be seen to consist entirely of minute spherules nearly approaching to an oval form. The cerebrum and cerebellum themselves, also approach nearly to the spherical and oval form, and thus assume a shape like that of their parts. Hence these minute organic substances, inasmuch as they are like their whole, and have the same potency individually, which, conjointly and aggregately, is exercised in the compound, merit the name of *cerebellula.* The eye also, by artificial aid, is enabled to discover that these forms, spherules, or cerebellula, are clothed with, and inclosed in, a membrane of meninx, much in the same manner as the brain itself, except that their membrane or meninx deserves the title of pia in the superlative degree, and that they are distinguished from their neighboring and associate spherules of the same kind. It may also be discerned, that these most delicate coats are composed of villi and capillary shoots, of most minute arteries in multitude innumerable, in determination wonderful, and in order most beautiful, which diffuse in all directions a volatile and spirituous fluid, educed from the blood, and conceived by eminent generation in their most pure wombs. These cerebellula appear to be the internal sensories, which receive impressions and modifications from the external sensories, and which convey them afterwards higher up to the judgment-seat of the mind." Vol 2, p. 41.

It would be useless to occupy the time of our readers with any further account of this work, which the editor implies is not so much designed for physiologists as "for the educated public ;" but assuredly the recollection that the peculiar views of Swedenborg, both as regards the constitution of matter in general and the phenomena of animal bodies, have commanded no attention; joined to the knowledge that the present volumes abound in errors as to matters of fact and observation, will indispose the judicious inquirer to place any reliance on hypotheses based upon such materials, and opposed, in so many respects, to the ascertained truths of physics and physiology.

